# Potential of Mesenchymal Stem Cell based application in Cancer

**Published:** 2015-04-01

**Authors:** Sushilkumar Ramdasi, Shabari Sarang, Chandra Viswanathan

**Affiliations:** Regenerative Medicine Group, Reliance Life Sciences Pvt. Ltd. Dhirubhai Ambani Life Sciences Centre, R-282, TTC Area of MIDC, Thane-Belapur Road, Rabale, Navi Mumbai - 400701. India

**Keywords:** Mesenchymal Stem Cells, Cancer, Anti-cancer, Tumor, Tropism

## Abstract

Stem cell based treatments are being increasingly explored for their possible potential to treat various cancers. Mesenchymal stem cells believed to possess anti-tumor potential and are preferred for their properties like immune privileged nature, ability to migrate to the site of tumor and capability for multilineage differentiation. This tumor tropism property of MSCs could be utilized to deliver anti-tumor biological agents to the site of tumor. In a tumor micro-environment, MSCs are believed to play both, a pro-tumorigenic and an anti-tumorigenic role. However, this is dependent on a host of factors like, types of MSCs, its source, type of cancer cell line under investigation, in vivo or in vitro conditions, factors secreted by MSCs and interactions between MSCs, host’s immune cells and cancer cells. Among several cytokines secreted by MSCs, TRAIL (Tumor necrosis factor related apoptosis inducing ligand) is reported to be pro-apoptotic for tumor cells.

The MSCs from bone marrow and adipose tissue have been studied quite extensively. Deriving MSCs from sources such as umbilical cord blood and umbilical cord tissue is relatively easier. Umbilical cord tissue preferred for MSC derivation due to their abundant availability. These MSCs believed to up regulate TRAIL expression in MSC-cancer cell co-culture system resulting in induction of apoptosis in cancer cells. However, umbilical cord tissue derived MSCs needs to be studied for expression pattern of TRAIL in a co-culture system.

We present a review article on different studies reporting both, pro-tumorigenic and anti-tumorigenic properties of MSCs.

## Introduction

 Cancer, known medically as a malignant neoplasm (new tissue), is a broad group of various diseases, involving unregulated cell growth. Cancer is generally treated with combination of therapies that include chemotherapy, radiotherapy, surgical resection, immunotherapy and monoclonal antibody therapy. The discovery of anti-cancer therapy in the mid-twentieth century was aimed at development of chemotherapeutic agents to target cancer cells.^1^ However; conventional cancer therapies (surgery, chemotherapy or radiotherapy) have their own limitations since these treatments do not deal with the challenge of tumor recurrence & metastasis following initial remission. Also, these conventional therapies lack precise specificity to target only cancer cells. This failure of cancer treatment is usually not contributed by lack of primary response or initial induction of remission, but by relapse or tumor recurrence after therapy, in which tumor-initiating cells might play a crucial role. There are several reports which demonstrate presence of tumor-initiating cells, popularly known as cancer stem cells, which support tumor growth and these cells can be targeted to eradicate cancer from its source.^2^^,^^3^^,^^4^ Tumor-initiating cells can be optimally defined as those tumor cells that have ability to re-grow the tumor from which they were isolated and identified. The term ‘cancer stem cell’ does not mean that the cell derived from normal stem cell.^5^ Rather, cancer stem cells produce tumors. The self-renewal and differentiation of cancer stem cells is maintained and regulated by its own niche. The traditional anti-cancer therapy targets only the differentiated tumor cells and not tumor initiating cells; by this consideration these treatments, to a significant extent, are limited to symptomatic treatments only. Recent advances in finding treatment options for cancer are exploring anti-cancer properties of human stem cells. It is the need of the hour to find out type of stem cells which are anti-cancer in nature by their interaction with tumor cells and its effect on tumor growth. We bring you a synopsis of such developments in the scientific community contributing to the possible cancer treatment using stem cells.

  **STEM CELLS**

  The discovery of stem cells in 1963 by E.A. McCulloch & J.E. Till opened infinite possibilities of use of these progenitor cells to treat various human illnesses.^6^^,^^7^ Their findings resulted into discovery of undifferentiated, self-renewing, progenitor cell population which are referred as stem cells. Stem cells, on the basis of their origin, can be divided into four broad categories; embryonic stem cells which are capable of a self-renewal and differentiation into cells of all tissue lineages;^8^ foetal stem cells which are located in foetal tissues and are multipotent in nature; adult stem cells are partially committed stem cells located in specific stromal niche and can be obtained from the mesodermal tissues such as BM,^9^ muscle,^10^ adipose tissue,^11^ synovium^12^ and periosteum^13^ and umbilical cord blood stem cells are divided into two sub-categories hematopoietic and mesenchymal stem cells.

 Of all these, the type of stem cells which are widely studied for their anti-cancer properties are mesenchymal stem cells (MSCs).^14^ MSCs are adult stem cells which were first identified in the stromal compartment of bone marrow by Friedenstein et al in 1960s.^15^^,^^16^

MSCs are preferred as these cells have the potential of multi lineage differentiation into bone, cartilage & fat cells,^17^ immune privilege nature,^18^ affinity for site of injury or tumor, ease of availability and maintenance in the culture and storage. The multi lineage differentiation potential of MSCs confer them crucial role in tissue regeneration and have been used experimentally to repair tissue damage in various disease conditions.^19^^,^^20^

 1990s is marked by the investigation of safety and efficacy of MSCs in clinical applications. The first study reported that MSCs are safe with complete remission in hematological malignancies.^21^ In this study 15 human subjects with hematological malignancies were infused with autologous cultured MSCs resulting into safe and complete remission. Another encouraging evidence about safety of MSCs came up when MSCs were successfully utilized to treat children affected by osteogenesis imperfecta.^22^ MSCs reported to be safe for application in heart degenerative diseases, inflammatory diseases and spinal cord injuries.^23^^,^^24^^,^^25^ MSCs from autologous origin were safe in treating liver disease,^26^ brain injury^27^ and myocardial infarction.^28^

 MSCs express major histocompatibility (MHC) class I surface markers and are negative for MHC class II expression. Also, they lack the co-stimulatory molecules CD40, CD80 and CD86. Despite expression of low levels of MHC class I antigens that results the activation of T cells, the absence of co-stimulatory molecules cannot initiate secondary signals, thus leaving the T cells anergic.^29^ Low levels of expression of MHC class I is important in protecting MSCs from natural killer cell-mediated cytotoxicity and cells that do not express MHC class I are targeted and destroyed.^30^ In fact, MSCs are immunosuppressive in nature^18^ which is the pre-requisite in allogeneic cell therapy based transplant.

  **TROPISM OF MSCs TOWARDS INJURY OR TUMOR**

 The discovery that mesenchymal stem cells (MSCs) are recruited into tumors has led to a great deal of interest over the past decade in the role of MSCs in tumors. This MSC tropism towards site of tumor has been put forth by Maestroni et al. in 1999,^31^ wherein they proved homing of MSCs to the tumors. The tumors are characterized as ‘wounds that do not heal’ and the MSC tropism to wounds is very well documented in many reports.^32^^,^^33^ MSCs interact with tumor cells in number of ways, which have the potential to support or suppress tumor growth. Report by Maestroni et al. (1999)^31^ has shown that bone marrow derived MSCs release some soluble factors that inhibit lung carcinoma and B16 melanoma growth in mice. Tropism of MSCs for glioma was first evidenced by implantation of rat MSCs into rats having syngeneic gliomas.^34^ It is also demonstrated that MSCs possess selective tropism for gliomas which is comparable with tropism by human neural stem cells (hNSC).^35^ The potential of hNSCs is limited by difficulties in harvesting and ex*-*vivo expansion of the same.^36^ In contrast, MSCs can be readily harvested and expanded from numerous sources without any practical difficulty as described above. As stated earlier tumor mimics a wound which serves as a continuous source of cytokines & chemokines^37^ thereby recruiting respondent cell types including MSCs. This homing potential of MSCs is evidenced in almost all tested human cancer cell lines^32^ such as melanoma,^38^ pancreatic cancer,^39^ ovarian cancer,^40^ colon cancer,^41^ breast cancer,^40^ lung cancer,^42^ Kaposi’s sarcoma^43^ and malignant glioma.^44^

 The probable mechanism of tumor-specific migration of MSCs is not fully understood, but seems to be dependent upon biological properties of tumor micro-environment.^45^ High concentrations of inflammatory chemokines & growth factors believed to be responsible for integration of MSCs into tumor stroma. Since tumor is considered a “wound”, its micro-environment is considered as a site of chronic inflammation.^37^ It is reported that it may be this micro-environment which mediates MSC migration through secretion of soluble factors such as epidermal growth factor, vascular endothelial growth factor-A, fibroblast growth factor, platelet-derived growth factor, stromal-derived growth factor-1α (SDF-1α/CXCL12), Interleukin-8 (IL-8), Interleukin-6 (IL-6), granulocyte–macrophage colony-stimulating factor, granulocyte colony-stimulating factor, Ang1, monocyte chemoattractant protein-1 (CCL2), hematopoietic growth factor, transforming growth factor beta-1 and urokinase-type plasminogen activator.^34^^,^^45^^,^^46^^,^^47^^,^^48^^,^^49^

 MSCs could be ideal delivery vehicles for anti-tumor biological agents owing to their properties like tumor tropism, integration into tumor stroma^38^ and immune privileged nature.^50^ Number of anti-tumor genes have been engineered into MSCs and successfully demonstrated anti-tumor effects on various cancer models, to list a few, IFN-ß on pancreatic cancer,^39^ IL-12 on melanoma and hepatoma,^51^ IFN-α on melanoma,^52^ IFN-r on leukemia,^53^ IL-2 on glioma,^34^ NK4 on lung cancer^54^ and TRAIL on breast cancer,^42^ glioma^44^ and lung cancer.^55^ MSC tropism is also reported to be utilized to deliver oncolytic viruses to tumor sites. Genetically modified oncolytic viruses such as Adenoviruses are tested as anti-tumor weapons. Study also confirmed that there is decrease in tumor burden in animals treated with oncolytic virus delivered by MSC carriers compared with the direct injection of the adenovirus.^56^

**MSCs – PRO-TUMORIGENIC OR ANTI-TUMORIGENIC?**

 There are other observations suggesting that, in the tumor micro-environment, MSCs have several tumor growth promoting functions, including expression of growth factors, promotion of tumor vessel formation and creation of tumor stem cell niches.^57^^,^^58^ It is believed that MSCs may contribute to tumor growth in several ways: (1) by promoting angiogenesis; (2) by creating a niche to support cancer stem cells survival; (3) by modulating the organism’s immune response against cancer cells; and (4) by promoting formation of metastasis.^59^ Through extensive literature survey, we observed that pro-tumorigenicity and anti-tumorigenicity of MSC on cancer probably depends on many factors which are explained in detail below. It appears that the source of MSCs is determinant of response to tumor; in one study researchers reported umbilical cord blood-derived MSCs causes brain tumor regression but adipose tissue-derived MSCs promotes the same.^60^ Tumor growth promotion is also reported in gastric cancer models,^61^ breast subcutaneous tumor models^62^ & ovarian cancer.^63^ Another study reports that melanoma cells lead to tumor formation only in the presence of MSCs when injected subcutaneously into an allogeneic recipient.^64^ Furthermore, tumor progression or regression is also dependant on in vitro or in vivo conditions.^14^ Tumor progression by MSCs were reported in melanoma A375 cells but was absent in glioblastoma 8MGGBA cell line,^65^ which is indicative of tumor cell specific behavior of MSCs. To investigate and understand whether advantages outweigh disadvantages of MSCs as therapeutic agent, it is imperative to review some more studies. Yet again, there are reports indicating MSCs as possibly the most promising delivery vehicle for a cell-based targeted cancer gene therapy^66^ & they serve as cytotoxic for certain type of cancer cells.^67^ In another study MSC conditioned media resulted in the downregulation of NFkB in hepatoma and breast cancer cells which decreased cancer cell in vitro proliferation.^68^ It has been reported that MSCs results into suppression of tumor growth in models of Kaposi’s sarcoma,^43^ Lewis lung carcinoma,^31^ glioma,^34^ colon carcinoma,^69^ malignant melanoma,^31^^,^^70^ breast cancer,^71^ pancreatic cancer^72^ and prostate carcinoma.^73^ Table 1 shows some of the in vivo experiments and possible mechanisms of action of MSCs to restrict tumor progression. It has been observed that bone marrow MSCs release soluble factors which shown significant reduction in tumor growth and progression in melanoma, lung carcinoma^31^ and in glioma models.^34^ The group of researchers reported umbilical cord blood-derived MSCs inhibits proliferation of Glioblastoma Multiforme by up-regulating secretion of TRAIL.^60^ The downregulation of Wnt signaling pathway in breast cancer reported to be related to secretion of dickkopf proteins (DKK-1).^68^ In another study, a completely novel approach was implemented by isolating two phenotypes of MSCs, called MSC1 and MSC2 from mixed population of MSC.^80^ Researchers in this study concluded that MSC1 is pro-inflammatory while MSC2 is immunosuppressive in nature. In continuation with this study, same group reported that MSC1 is anti-tumorigenic while MSC2 is pro-tumorigenic in nature.^81^ Wnt pathway plays a crucial role in stem cell self-renewal and differentiation. It has been reported that human tumor progression is a result of an aberrant Wnt pathway.^74^ The evidence that MSCs inhibit Wnt pathway reported by study wherein researchers co-cultured MSCs with human hepatoma cells (H7420) that resulted in decreased cell proliferation, increased apoptosis and downregulation of all target genes of Wnt signaling (Bcl-2, c-Myc, PCNA and Survivin). The results were obtained when MSC conditioned medium was used, which is indicative of involvement of paracrine effect. As can be seen, mechanisms of anti-tumor properties of MSCs are not fully elucidated and more insights are awaited, it is presumed that it has a strong relation to downregulation of Akt, NFkB & Wnt signaling pathways.^75^ Stem cells and tumor cells have similar signaling pathways regulating self-renewal and differentiation, including Wnt, Notch, Shh and BMP pathways.^76^^,^^77^^,^^78^ Stem cells possess strong regulatory control over these pathways, while tumor cells do not possess the same. Loss of regulation of pathways involved in multiplication leaves tumor cells immortal and they multiply without any control.

 Multiple mechanisms have been postulated to be contributing to anti-tumor potential of MSCs. Few of the probable mechanisms include apoptosis caused due to upregulation of TRAIL, cell cycle arrest, direct cytokine mechanism, blocking of PI3K/ AKT pathway, expression of tumor suppressor genes, downregulation of Wnt pathway and expression of DKK1. 

## CONCLUSION

 Discovery of stem cells paved its way in making new cell therapy options available to patients suffering from various diseases. The therapeutic potential of stem cells is being extensively studied for the possible treatment of degenerative diseases and lifestyle disorders ranging from autoimmune diseases, multiple sclerosis, diabetes mellitus, ischemic diseases like stroke, heart failure, cartilage repair, liver cirrhosis, neurological disorders like Parkinson’s disease, Huntington’s disease and spinal cord lesions. Recently, yet another application of stem cells is being explored in an attempt to find out possible cell therapy for various malignancies like renal cell cancer, breast cancer, lung cancer, ovarian cancer, leukemia, melanoma, pancreatic cancer, malignant glioma and so on.

**Table 1:** Probable anti-cancer mechanism of MSCs on different animal models of cancer- Summary of few in vivo experiments using different animal models of cancer indicating MSC source, type of animal model, mode of MSC infusion, observations and probable mechanism of action involved in tumor regression

**Reference**	**Otsu 2009** ^70^	**Doi 2010** ^72^	**Cousin2009** ^79^	**Chanda 2009** ^73^	**Ren 2008** ^52^
**Observations**					
**Cancer**	Melanoma	Pancreatic cancer	Pancreatic cancer	Prostate cancer	Prostate cancer
**Cancer cell type**	B16F10	PAN02	Capan-1	Osteolytic human prostate cancer cells PC3 expressing firefly luciferase	Mouse metastatic prostate cancer cell line TRAMP-C2
**MSC source**	Bone marrow	Umbilical Cord Tissue	Adipose tissue	Bone marrow	Bone marrow: Undifferentiated BMMSCs tranduced with 1000 MOI of rAAV6-IFN-β or rAAV6-GFP
**Animal model**	Subcutaneous graft in C57/BL mice	Orhtotopicxenograft in C57/BL mice	Subcutaneous xenograft in athymic nude mice	Male SCID mice with intratibial injection	Prostate cancer cells lung metastasis in male C57BL/6 mice
**Mode of MSC infusion**	IT injection	IP injection	IT injection	Intratibial injection	IV injection
**Observations**	Regression in tumor size	Regression of tumor size with enhancement in survival time	Regression in tumor size	Inhibition of tumor growth	Regression in tumor growth
**Possible mode of action**	Apoptosis induction and angiogenesis inhibition	Interference with proliferation and G0/G1 arrest	Interference with proliferation and G1 arrest	New bone formation around tumor cells in tibia restricted growth of prostate cancer cells	Tumor apoptosis, anti-angiogenesis and induction of NK cell activity

 MSCs could be the next candidate of choice being considered for the treatment regime of cancer; especially in allogeneic mode they could possess tropism for solid tumors, immune privileged nature, ease of availability and maintenance etc. Application of MSCs for the possible treatment of tumors has divided the scientific society into two schools of thoughts. One school of thought states MSCs plays crucial role in tumorigenesis by promoting angiogenesis, creating a niche to support cancer stem cells survival, modulating the organism’s immune response against cancer cells and promoting formation of metastasis. The other school demonstrated anti-tumor effect of MSCs in few specific cancers.

 The exact mechanisms of anti-tumor property of MSCs are yet to be understood. However, predominant studies relate it with downregulation of Akt, NFkB & Wnt signaling pathways.

The source of MSC seems to contribute to anti-tumor properties against particular malignancy. Anti-tumor effect of umbilical cord blood derived MSCs was evident against brain tumor but the same effect was missing with MSCs derived from adipose tissue. The reasons for this can be a subject of new research. 

 Majority of MSCs studied for anti-tumor properties are derived from sources like bone marrow and adipose tissue. However, there are very few reports of usage of umbilical cord tissue as a source of MSCs. Umbilical cord tissue is a rich source of MSCs, the extraction procedure is relatively non-invasive, there are not ethical concerns and are available in abundance. The MSCs derived from umbilical cord tissue needs to be studied further for their anti-tumor properties in detail.

 This is a very interesting area that is worth following closely. Physicians, research community and others involved in cancer treatment will look forward to this new dimension with great deal of interest and the space is worth watching for new developments in near future.

## References

[1] Gilman A (1963). The initial clinical trial of nitrogen mustard. Am J Surg.

[2] Singh SK, Hawkins C, Clarke ID (2004). Identification of human brain tumour-initiating cells. Nature.

[3] O’Brien CA, Pollett A, Gallinger S (2007). A human colon cancer cell capable of initiating tumour growth in immunodeficient mice. Nature.

[4] Li C, Heidt DG, Dalerba P (2007). Identification of pancreatic cancer stem cells. Cancer Res.

[5] Clarke MF, Dick JE, Dirks PB (2006). Cancer stem cells — perspectives on current status and future directions: AACR Workshop on cancer stem cells. Cancer Res.

[6] Becker AJ, McCulloch EA, Till JE (1963). Cytological demonstration of the clonal nature of spleen colonies derived from transplanted mouse marrow cells. Nature.

[7] Siminovitch L, McCulloch EA, Till JE (1963). The distribution of colony-forming cells among spleen colonies. Journal of Cellular and Comparative Physiology.

[8] Pessina A, Gribaldo L (2006). The key role of adult stems cells: therapeutic perspectives. Curr Med Res Opin.

[9] Pittenger MF, Mackay AM, Beck SC (1999). Multilineage potential of adult human mesenchymal stem cells. Science.

[10] Deasy BM, Li Y, Huard J (2004). Tissue engineering with muscle-derived stem cells. Curr Opin Biotechnol.

[11] Zuk PA, Zhu M, Mizuno H (2001). Multilineage cells from human adipose tissue: implications for cell-based therapies. Tissue Eng.

[12] De Bari C, Dell'Accio F, Tylzanowski P (2001). Multipotent mesenchymal stem cells from adult human synovial membrane. Arthritis Rheum.

[13] Zarnett R, Salter RB (1989). Periosteal neochondrogenesis for biologically resurfacing joints: its cellular origin. Can J Surg.

[14] Tian LL, Yue W, Zhu F Human Mesenchymal Stem Cells Play a Dual Role on Tumor Cell Growth In Vitro and In Vivo.

[15] Friedenstein AJ, Piatetzky-Shapiro II, Petrakova KV (1966). Osteogenesis in transplants of bone marrow cells. J Embryol Exp Morphol.

[16] Friedenstein AJ, Petrakova KV, Kurolesova AI (1968). Heterotopic of bone marrow. Analysis of precursor cells for osteogenic and hematopoietic tissues. Transplantation.

[17] Cooper K, Sen Majumdar A, Viswanathan C (2010). Derivation, expansion and characterisation of clinical grade Mesenchymal stem cells from umbilical cord matrix using cord blood serum. International journal of stem cells..

[18] Tipnis S, Viswanathan C, SenMajumdar A (2010). Immunosuppressive Properties of Human Umbilical Cord Derived Mesenchymal Stem Cells: Role of B7-H1 and IDO. Immunology and Cell Biology Journal.

[19] Dominici M, Le Blanc K, Mueller I (2006). Minimal criteria for defining multipotent mesenchymal stromal cells. The International Society for Cellular Therapy position statement. Cytotherapy.

[20] Prockop DJ (2009). Repair of tissues by adult stem/progenitor cells (MSCs): controversies, myths, and changing paradigms. Mol Ther.

[21] Lazarus HM, Haynesworth SE, Gerson SL (1995). Ex vivo expansion and subsequent infusion of human bone marrow-derived stromal progenitor cells (mesenchymal progenitor cells): implications for therapeutic use. Bone Marrow Transplantation.

[22] Horwitz EM, Prockop DJ, Lorraine A (1999). Transplantability and therapeutic effects of bone marrow-derived mesenchymal cells in children with osteogenesis imperfecta. Nature Medicine.

[23] Le Blanc K, Rasmusson I, Sundberg B (2004). Treatment of severe acute graft-versus-host disease with third party haploidentical mesenchymal stem cells. Lancet.

[24] Le Blanc K, Frassoni F, Ball L (2008). Mesenchymal stem cells for treatment of steroid-resistant, severe, acute graft-versus host disease: a phase II study. Lancet.

[25] Chen S, Liu Z, Tian N (2006). Intracoronary transplantation of autologous bone marrow mesenchymal stem cells for ischemic cardiomyopathy due to isolated chronic occluded left anterior descending artery. Journal of Invasive Cardiology.

[26] Dai LJ, Li HY, Guan LX (2009). The therapeutic potential of bone marrow-derived mesenchymal stem cells on hepatic cirrhosis. Stem Cell Res.

[27] Zhang ZX, Guan LX, Zhang K (2008). combined procedure to deliver autologous mesenchymal stromal cells to patients with traumatic brain injury. Cytotherapy.

[28] Strauer BE, Brehm M, Zeus T (2005). Regeneration of human infarcted heart muscle by intracoronary autologous bone marrow cell transplantation in chronic coronary artery disease: the IACT Study. J Am CollCardiol.

[29] Javazon EH, Beggs KJ, Flake AW (2004). Mesenchymal stem cells: paradoxes of passaging. Experimental Hematology.

[30] Moretta A, Bottino C, Vitale M (2001). Activating receptors and coreceptors involved in human natural killer cell mediated cytolysis. Annual Review of Immunology.

[31] Maestroni GJ, Hertens E, Galli P (1999). Factors from nonmacrophage bone marrow stromal cells inhibit Lewis lung carcinoma and B16 melanoma growth in mice. Cell Mol Life Sci.

[32] Sun XY, Nong J, Qin K (2011). Mesenchymal stem cell-mediated cancer therapy: A dual-targeted strategy of personalized medicine. World J Stem Cells.

[33] Pendleton C, Li Q, Chesler DA (2013). Mesenchymal Stem Cells Derived from Adipose Tissue vs Bone Marrow: In Vitro Comparison of Their Tropism towards Gliomas. PLoS ONE.

[34] Nakamura K, Ito Y, Kawano Y (2004). Antitumor effect of genetically engineered mesenchymal stem cells in a rat glioma model. Gene Ther.

[35] Kosztowski T, Zaidi HA, Quinones-Hinojosa A (2009). Applications of neural and mesenchymal stem cells in the treatment of gliomas. Expert Rev Anticancer Ther.

[36] Lamfers M, Idema S, van Milligen F (2009). Homing properties of adipose-derived stem cells to intracerebral glioma and the effects of adenovirus infection. Cancer Lett.

[37] Dvorak HF (1986). Tumors: wounds that do not heal. Similarities between tumor stroma generation and wound healing. N Engl J Med.

[38] Studeny M, Marini FC, Champlin RE (2002). Bone marrow-derived mesenchymal stem cells as vehicles for interferon-beta delivery into tumors. Cancer Res.

[39] Kidd S, Caldwell L, Dietrich M (2010). Mesenchymal stromal cells alone or expressing interferon-beta suppress pancreatic tumors in vivo, an effect countered by anti-inflammatory treatment. Cytotherapy.

[40] Kidd S, Spaeth E, Dembinski JL (2009). Direct evidence of mesenchymal stem cell tropism for tumor and wounding micro-environments using in vivo bioluminescent imaging. Stem Cells.

[41] Menon LG, Picinich S, Koneru R M (2007). Differential gene expression associated with migration of mesenchymal stem cells to conditioned medium from tumor cells or bone marrow cells. Stem Cells.

[42] Loebinger MR, Kyrtatos PG, Turmaine M (2009). Magnetic resonance imaging of mesenchymal stem cells homing to pulmonary metastases using biocompatible magnetic nanoparticles. Cancer Res.

[43] Khakoo AY, Pati S, Anderson SA (2006). Human mesenchymal stem cells exert potent antitumorigenic effects in a model of Kaposi’s sarcoma. J Exp Med.

[44] Sasportas LS, Kasmieh R, Wakimoto H (2009). Assessment of therapeutic efficacy and fate of engineered human mesenchymal stem cells for cancer therapy. Proc Natl Acad Sci USA.

[45] Roisin M D, Sonja K, Frank P B (2010). Advances in mesenchymal stem cell-mediated gene therapy for cancer. Stem Cell Research & Therapy.

[46] Nakamizo A, Marini F, Amano T (2005). Human bone marrow-derived mesenchymal stem cells in the treatment of gliomas. Cancer Res.

[47] Studeny M, Marini FC, Dembinski JL (2004). Mesenchymal stem cells: potential precursors for tumor stroma and targeted-delivery vehicles for anticancer agents. J Natl Cancer Inst.

[48] Wels J, Kaplan RN, Rafi i S (2008). Migratory neighbors and distant invaders: tumor-associated niche cells. Genes Dev.

[49] Dwyer RM, Potter-Beirne SM, Harrington KA (2007). Monocyte chemotactic protein-1 (MCP-1) secreted by primary breast tumors stimulates migration of mesenchymal stem cells (MSCs). Clin Cancer Res.

[50] O’Donoghue K, Chan J, Fuente J (2004). Microchimerism in female bone marrow and bone decades after fetal mesenchymal stem-cell trafficking in pregnancy. Lancet.

[51] Chen X, Lin X, Zhao J (2008). A tumor-selective biotherapy with prolonged impact on established metastases based on cytokine gene-engineered MSCs. Mol. Ther.

[52] Ren C, Kumar S, Chanda D (2008). Therapeutic potential of mesenchymal stem cells producing interferon-alpha in a mouse melanoma lung metastasis model. Stem Cells.

[53] Li X, Lu Y, Huang W (2006). In vitro effect of adenovirus-mediated human Gamma Interferon gene transfer into human mesenchymal stem cells for chronic myelogenous leukemia, Hematol. Oncol.

[54] Kanehira M, Xin H, Hoshino KT (2007). Targeted delivery of NK4 to multiple lung tumors by bone marrow-derived mesenchymal stem cells. Cancer Gene Ther.

[55] Grisendi G, Bussolari R, Cafarelli L (2010). Adipose-derived mesenchymal stem cells as stable source of tumor necrosis factor related apoptosis-inducing ligand delivery for cancer therapy. Cancer Res.

[56] Komarova S, Kawakami Y, Stoff-Khalili MA (2006). Mesenchymal progenitor cells as cellular vehicles for delivery of oncolytic adenoviruses. Mol Cancer Ther.

[57] Zhu W, W Xu, R Jiang (2006). Mesenchymal stem cells derived from bone marrow favor tumor cell growth in vivo. Exp Mol Pathol.

[58] Yu JM, ES Jun, YC Bae (2008). Mesenchymal stem cells derived from human adipose tissues favor tumor cell growth in vivo. Stem Cells Dev.

[59] Galderisi U, Giordano A, Paggi Marco G (2010). The bad and the good of mesenchymal stem cells in cancer: Boosters of tumor growth and vehicles for targeted delivery of anticancer agents. World J Stem Cells.

[60] Keiko A, Kenichi K, Masumi N (2013). Umbilical Cord Blood-Derived Mesenchymal Stem Cells Inhibit, But Adipose Tissue-Derived Mesenchymal Stem Cells Promote, Glioblastoma Multiforme Proliferation. STEM CELLS AND DEVELOPMENT.

[61] Okumura T, Wang SS, Takaishi S (2009). Identification of a bone marrow-derived mesenchymal progenitor cell subset that can contribute to the gastric epithelium. Lab. Invest.

[62] Karnoub AE, Dash AB, Vo AP (2007). Mesenchymal stem cells within tumour stroma promote breast cancer metastasis. Nature.

[63] R Lis, C Touboul, P Mirshahi (2010). Tumor associated mesenchymal stem cells protects ovarian cancer cells from hyperthermia through CXCL12. Int. J. Cancer.

[64] Djouad F, Plence P, Bony C (2003). Immunosuppressive effect of mesenchymal stem cells favors tumor growth in allogeneic animals. Blood.

[65] Kucerova L, Matuskova M, Hlubinova K (2010). Tumor cell behaviour modulation by mesenchymal stromal cells. Molecular Cancer.

[66] Roorda BD, Elst A, Kamps WA (2009). Bone marrow-derived cells and tumor growth: Contribution of bone marrow-derived cells to tumor micro-environments with special focus on mesenchymal stem cells. Crit Rev Oncol Hematol.

[67] Kang SG, Jeun SS, Lim JY (2008). Cytotoxicity of human umbilical cord blood-derived mesenchymal stem cells against human malignant glioma cells. Childs Nerv Syst.

[68] Qiao L, Zhao TJ, Wang FZ (2008). NFkappa B downregulation may be involved the depression of tumor cell proliferation mediated by human mesenchymal stem cells. Acta Pharmacol Sin.

[69] Ohlsson LB, Varas L, Kjellman C (2003). Mesenchymal progenitor cell-mediated inhibition of tumor growth in vivo and in vitro in gelatin matrix. Exp Mol Pathol.

[70] Otsu K, Das S, Houser SD (2009). Concentration-dependent inhibition of angiogenesis by mesenchymal stem cells. Blood.

[71] Ayuzawa R, Doi C, Rachakatla RS (2009). Naive human umbilical cord matrix derived stem cells significantly attenuate growth of human breast cancer cells in vitro and in vivo. Cancer Lett.

[72] Doi C, Maurya DK, Pyle MM, Tamura M (2010). Cytotherapy with naive rat umbilical cord matrix stem cells significantly attenuates growth of murine pancreatic cancer cells and increases survival in syngeneic mice. Cytotherapy.

[73] Chanda D, Isayeva T, Kumar S (2009). Therapeutic Potential of Adult Bone Marrow–Derived Mesenchymal Stem Cells in Prostate Cancer Bone Metastasis. Clin Cancer Res.

[74] Chen G, Shukeir N, Potti A (2004). Up-regulation of Wnt-1 and beta-catenin production in patients with advanced metastatic prostate carcinoma: potential pathogenetic and prognostic implications. Cancer.

[75] Loebinger MR, Janes SM (2010). Stem cells as vectors for antitumour therapy. Thorax.

[76] Reya T, Clevers H (2005). Wnt signalling in stem cells and cancer. Nature.

[77] Duncan AW, Rattis FM, DiMascio LN (2005). Integration of Notch and Wnt signaling in hematopoietic stem cell maintenance, Nat. Immunol.

[78] Androutsellis-Theotokis A, Leker RR, Soldner F (2006). Notch signalling regulates stem cell numbers in vitro and in vivo. Nature.

[79] Cousin B, Ravet E, Poglio S (2009). Adult stromal cells derived from human adipose tissue provoke pancreatic cancer cell death both in vitro and in vivo. PLoS One.

[80] Waterman RS, Tomchuck SL, Henkle SL (2010). A new mesenchymal stem cell (MSC) paradigm: polarization into a pro-inflammatory MSC1 or an Immunosuppressive MSC2 phenotype. PLoS One.

[81] Waterman RS, Henkle SL, Betancourt AM (2012). Mesenchymal Stem Cell 1 (MSC1)-Based Therapy Attenuates Tumor Growth Whereas MSC2-Treatment Promotes Tumor Growth and Metastasis. PLoS One.

[82] Shuichi M, Ting Y, Akifumi M (2014). Cancer stem cells maintain a hierarchy of differentiation bycreating their niche. Int. J. Cancer.

